# Determination of the Masking Effect of the ‘Zapateria’ Defect in Flavoured Stuffed Olives Using E-Nose

**DOI:** 10.3390/molecules27134300

**Published:** 2022-07-04

**Authors:** Ramiro Sánchez, Emanuele Boselli, Antonio Fernández, Patricia Arroyo, Jesús Lozano, Daniel Martín-Vertedor

**Affiliations:** 1 Technological Institute of Food and Agriculture CICYTEX-INTAEX, Junta of Extremadura, Avda. Adolfo Suárez s/n, 06007 Badajoz, Spain; ramiro.sanchez@juntaex.es (R.S.); antonio.fernandezf@juntaex.es (A.F.); 2 Faculty of Science and Technology, Free University of Bozen-Bolzano, Piazza Università 1, 39100 Bozen-Bolzano, Italy; 3 Industrial Engineering School, University of Extremadura, 06006 Badajoz, Spain; parroyoz@unex.es (P.A.); jesuslozano@unex.es (J.L.); 4 Research Institute of Agricultural Resources (INURA), Campus Universitario, Avda. de la Investigación s/n, 06071 Badajoz, Spain

**Keywords:** sensory analysis, stuffed olives, electronic nose, ‘Zapateria’ defect, ‘Mojo picón’ flavour, aromas

## Abstract

Spanish-style table olives are one of the most common processed foods in the Mediterranean countries. Lack of control during fermentation can lead to one of the main defects of the olive, called ‘Zapateria’, caused by the combination of volatile fatty acids reminiscent of rotten leather. In this study, table olives altered with ‘Zapateria’ defect were stuffed with a hydrocolloid flavoured with the aroma ‘Mojo picón’ to improve consumer acceptance. Sensory analysis, determination of volatile compounds and electronic nose (E-nose) were used to evaluate the quality of the olives. The control samples had a high concentration of the defect ‘Zapateria’ and were classified in the second commercial category, while higher ‘Mojo picón’ flavour concentrations resulted in these olives being classified as ‘extra category’ (a masking effect). The main volatile compounds in olives with ‘Zapateria’ defect were cyclohexanecarboxylic acid and pentanoic acid. E-nose allowed discrimination between stuffed olives without added flavouring and olives with ‘Mojo picón’ flavouring at different concentrations. Finally, PLS regression allowed a predictive linear model to be established between E-nose and sensory analysis values. The $R_{P}^{2}$ values were 0.74 for perceived defect and 0.86 for perceived aroma. The E-nose was successfully applied for the first time to classify Spanish-style table olives with ‘Zapateria’ defect intensity and with the addition of the ‘Mojo picón’ aroma masking the defect.

## 1. Introduction

According to the International Olive Oil Council [1], world table olive production is expected to increase by 7% in the 2021/2022 season, reaching 2,846,500 tonnes. Spain stands out as the world’s largest producer and one of the countries with the highest increase in production (18%), reaching 645,000 tonnes, compared to the average production of 551,000 tonnes. Regarding consumption for 2021/2022, the IOC forecasts a 1.2% increase compared to 2020/2021. Table olives are widely consumed in the Mediterranean coast and worldwide and are elaborated according to different styles.

One of the most common products is known as “green olives treated in brine in the Sevillian or Spanish style” [2,3]. Spanish-style processing consists of an alkaline treatment with NaOH (1.5–4.5%) to remove bitterness, followed by several washes with water to remove residual lye, and then fermentation in brine to improve the nutritional and sensory quality of the product. With the alkaline treatment, the pH of the olive pulp reaches values of 11.0–13.0, which decrease to 8.0–9.0 after repeated washings. After washing, the olives are immersed in 6–10% NaCl [4]. At the end of the lactic fermentation (when glucose and reducing sugars are exhausted), the pH drops to 4.0 or less; this increase in acidity ensures the preservation of the product. At the end of this phase, if the product is not pasteurised, it can undergo undesirable fermentation during storage, which can lead to an increase in pH and volatile acidity, a decrease in lactic acid, the formation of cyclohexanecarboxylic acid [5] and the production of biogenic amines, such as cadaverine and tyramine [6]. In order to avoid uncontrolled and harmful microbial growth, the pH must be kept <4.0 and the NaCl in the brine must be raised to >8% [4].

During the elaboration, optimal processing conditions preventing microbial spoilage must be applied. Numerous works have been published on olives processed according to the “Spanish style” [7,8,9,10]. When fermented olives are not produced according to the best practices, an outbreak of putrefaction called ‘Zapateria’ can occur. Among the volatile compounds responsible for the unpleasant smell of the ‘Zapateria’ off-flavour is cyclohexanecarboxylic acid [5]. Other chemical compounds related to the ‘Zapateria’ defect and formed in fermented green table olives during storage are propionic acid (from microorganisms of the genus *Propionibacterium*), volatile acids such as formic, butyric, succinic, isobutyric and n-valeric acid, as well as acetaldehyde, methanol, ethanol, 2-butanol and n-butanol. *Clostridium* species related to the final steps of ‘Zapateria’ deterioration have also been found [6].

For the present study, the raw material used were “Spanish-style” green olives affected by ‘Zapateria’ defect and filled with hydrocolloids to which ‘Mojo picón’ flavouring was added (‘Mojo picón’ is the flavour of a hot sauce prepared with garlic, cumin, sweet paprika, chilli pepper, sea salt, olive oil and vinegar). The green olives are those obtained from fruits harvested before veraison when they have reached their final size. In the table olives industry, stuffed table olives can be prepared in different ways. After being pitted, olives can be stuffed with pepper, onion, almonds, celery, anchovy, orange or lemon peel, hazelnut, caper or with their pastes prepared for stuffing [11].

For decades, stuffing has usually consisted of a paste with an indeterminate amount of flavouring ingredients or additives, such as a piece of anchovy and pepper. This is an expensive and very laborious procedure, so stuffed olives may also contain spices and aromatic herbs or their natural extracts and authorised additives, including flavourings, thickeners and binders for food use as defined by the Codex Alimentarius for this product and with limited use according to Good Manufacturing Practices [12,13]. The filling consists of a gelled mass based on alginate, a stabiliser that holds the different components of the mass together and prevents them from escaping from the inside of the olives. It should be noted that the use of hydrocolloids in food is becoming increasingly common. They can influence food processing, nutritional and sensory properties, leading to benefits in the final product [14]. In this regard, new electronic and digital technologies that aim to better understand different food matrices should be considered.

Recently, an electronic nose has been used to evaluate abnormal fermentation defects in “Spanish-style” table olives, including the defects of ‘Zapateria’, ‘Butyric’, ‘Putrid’ and ‘Mould’ [15,16]. The “Spanish style” processed green olives used in this study had the ‘Zapateria’ defect. Inoculating the brine with selected starter cultures (lactic acid bacteria and yeasts) reduces the probability of spoilage and helps to achieve a better and more predictable fermentation process [4]. Therefore, the control of fermentation processes can be performed by monitoring the pH and NaCl concentration of the brine, chromatographic detection of volatile organic compounds (VOC), microbiological analysis and also organoleptic evaluation [1]. According to the IOC method, table olives are to be commercially graded taking into account the intensity of any defects determined by a panel of 8 to 12 tasters, based on smell, taste and texture. At the same time, the IOC method defines the characteristic olfactory sensations of abnormal fermentations in table olives. These include the negative attribute of ‘Zapateria’, caused by the combination of volatile fatty acids reminiscent of rotten leather.

The electronic nose (E-nose) is a low-cost device made from a combination of sensors that allows samples with different aroma profiles to be discriminated in different types of matrices. In the wine matrix, its use has been studied to avoid the development of unpleasant flavours and aromas in final wines [17]; in edible oils, it has been used for the qualitative evaluation of the storage period [18]; in citrus fruits, for the detection of infections [19]; in garlic, for the early detection and classification of fungal infections [20]. This device has also been used to classify the quality of olives from different olive trees [21] or even to differentiate table olives with abnormal fermentations [15,16]. In this sense, the E-nose, due to its speed and low cost, can be an alternative to chromatography and sensory panel to assess the presence of defects. This is an important aspect for the subsequent classification of the product according to its flavour profile.

For these reasons, the main objective of this work was to study the olfactory evolution of table olives with a ‘Zapateria’ defect by means of E-nose, before and after the addition of different concentrations of ‘Mojo picón’ aroma to the filling. The aim was to show that the ‘Zapateria’ defect can be masked by a certain concentration of added flavours, which improve consumer acceptance.

## 2. Results and Discussion

### 2.1. Sensory Aroma of Hydrocolloid-Filled Olives

A trained panel was used to determine the ‘Mojo picón’ aroma intensity and the negative odour related to ‘Zapateria’ of ‘Spanish style table olives (variety ‘Manzanilla de Sevilla’) stuffed with different concentration of hydrocolloids (M2, M4 and M8) (Table 1).

Firstly, it should be noted that the control sample had a high concentration of ‘Zapateria’ defect. Therefore, according to the IOC regulations, from the results obtained, the olives can be classified into different sensory categories based on the evaluation of the defect predominantly perceived (DPP) by the tasting panel [1]. Therefore, the control samples were classified in the second category (Table 1). This defect is characteristic of this type of table olive processing, so an attempt was made to see how this defect was masked by the addition of different flavour concentrations. Flavouring was added at different concentrations in the hydrocolloids, except in the control samples, where no aroma was added.

The results showed that the aroma concentration of the table olive stuffing significantly influenced the aroma intensity and defect perception of the olives studied (*p*-value < 0.05).

In other words, the tasters were able to discriminate the different samples according to the intensity of the flavour added (Table 1). The intensity of the ‘Mojo picón’ flavour ranged from 3.1 (M2) to 7.2 (M8). This was a good result since the application of this flavour concentration to the hydrocolloids was sufficient to obtain a very intense aroma in whole olives.

On the other hand, the perception of the intensity of the defect by the tasters decreased as the concentration of ‘Mojo picón’ flavouring increased. However, the defective olives were flavoured with different concentrations which resulted in a decrease in the intensity of the ‘Zapateria’ defect in the flavoured stuffed olives. The defect ranged from 3.5 (M2) until not being detected (M8). Therefore, these olives were classified in the best commercial category called ‘extra category’ or ‘fancy’ (DPP ≤ 3) when the olives were stuffed with M4 and M8. It should be noted that the defect was perceived more in this type of olives than in those with a lower concentration of aroma (M2), classified as ‘first category’ or ‘selected’ (3 < DPP ≤ 4.5). Finally, the olives without aroma had a high defect intensity, being classified as ‘second category’ or ‘standard’. It can, therefore, be stated that the intensity of the aroma of the filling caused a decrease in the perception of the ‘Zapateria’ defect in the studied samples and this could serve to mask the defect and market the olives filled with hydrocolloids in a better commercial category.

It must be considered that the olives without flavouring presented a significantly more intense defect, corresponding to a worse category, than the olives stuffed with the flavoured hydrocolloids. Furthermore, the olives with the highest flavour concentration presented a significantly lower defect intensity, being classified as olives with a higher commercial category (DPP ≤ 3). The results show that stuffing the olives with different concentrations of flavoured hydrocolloids could be a strategy to mask the ‘Zapateria’ defect, making Spanish-style green olives more attractive to the consumer. This aroma of ‘Mojo picón’ was previously used in the stuffed black olives by Sánchez et al. [22]. This flavour had a good aromatic intensity compared to other flavourings used and it was enough to mask the “cooked effect” in “California-style” black olives. In addition, this filling presented an intense orange colour that stands out with the black and green table olives.

### 2.2. Volatile Compounds of Hydrocolloid-Filled Olives

Volatile compounds were analysed in table olives stuffed with the aromatic hydrocolloid at different concentrations (M2–M8). The identified volatile compounds listed and their percentage content of the odor are shown in Table 2. Of all the volatile compounds detected by gas chromatography, the 16 most representative volatile compounds of table olives were determined. Of these, 8 compounds were found in the pure flavour, 8 in table olive altered with ‘Zapateria’ defect (C) and 9 in table olives stuffed with different concentrations of ‘Mojo picón’ flavour (M2, M4 and M8).

The main constituents of the volatile matrix in pure aroma were beta-pinene (19.7%), p-cymene (18.7%), gamma-terpinene (24.7%) and diallyl disulphide (14.6%). The aroma of these compounds is related to positive attribute such as fresh fruit, citrus, herbs and green onions. The main constituents of the green olives with ‘Zapateria’ defect were creosol (25.0%), propylene glycol (19.8%), 2,4-hexadienoic acid, ethyl ester (14.5%), cyclohexancarboxylic acid (12.7%) and 2.4-hexadienoic acid methyl ester (11.2%). The volatile compounds responsible for unpleasant odors were propylene glycol, cyclohexanecarboxylic acid, pentanoic acid and 2-decenal, (E)-. These compounds were not found at high concentrations, but they are related to ‘Zapateria’ spoilage [8,19,20]. Cyclohexanecarboxylic acid and pentanoic acid have been described by Sanchez et al. [15,22] as the molecules responsible for the unpleasant odor of ‘Zapateria’ of Spanish-style table olives. It should be noted that the odor of pentanoic acid is also related to the butyric defect as reported by previous studies [15].

However, these compounds decreased considerably in the altered olives, and other compounds appeared. Filling of defective olives with hydrocolloid caused the dilution of the aroma compounds linked to the olive defects, regardless of the concentration used. This effect was clearly observed even in the aroma at low concentration. The predominant volatile compounds in olives filled with aromatised hydrocolloid were practically the same as those present in the pure flavour of ‘Mojo picón’, but these aromas were at different concentrations.

### 2.3. Application of E-nose for the Discrimination of Stuffed Olives

The information obtained from the E-nose has multiple variables and, in order to be able to interpret them, it is necessary to reduce them as much as possible. Using Principal Component Analysis (PCA) we can go from 11 variables, one for each sensor, to two or three principal components (linear combination of variables). The Principal Components allow the graphical representation of the grouping of data with similar values.

**Figure 1:**
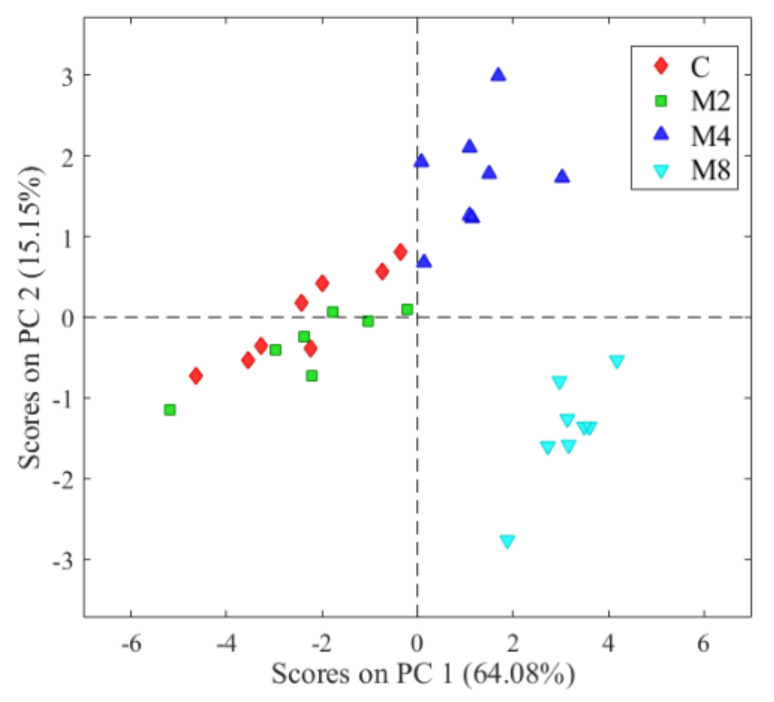
Score plot obtained from the PCA of olives stuffed with flavoured hydrocolloids at different concentrations. C: Spanish-style green table olives without aroma; M2: Spanish-style green table olives with ‘Mojo picón’ flavour at 2%; M4: Spanish-style green table olives with ‘Mojo picón’ flavour at 4%; M8: Spanish-style green table olives with ‘Mojo picón’ flavour at 8%. n.d.: not detected.

PCA in E-nose applications has been widely used in food quality control [23], classification and quality control of edible oils [24] and the half-life of vegetable oils [25]. The unsupervised exploratory PCA, based on the first two components, was able to differentiate between stuffed olives without added flavouring and olives added with ‘Mojo picón’ flavouring at different concentrations (Figure 1). PC1 explained 64.0% of the total variance of the data, while PC2 explained 15.1%. These results coincide with those obtained by Sánchez et al. [22], who were able to separate stuffed black olives with and without added flavouring. In Figure 1, the scores close to each other represent observations with similar characteristics. There is proximity of the red points (C) and green points (M2). This indicates that ’Mojo picón’ aroma added at a low concentration (2%) is not enough to fully achieve the objective of masking the initial aroma of table olives with a predominant ’Zapateria’ defect. On the contrary, the light blue points (M8) with high concentration (8%) are further away from the control (C, red points) showing that the defect was efficiently masked by 8% ’Mojo picón’ aroma. This evidence could be interesting for table olives producers to obtain a better product without organoleptic defects.

Subsequently, a classification analysis was performed by PLS-DA using leave-one-out cross-validation. The PLS-DA algorithm has previously been used to classify post-harvest olive fruit quality [21] and discrimination of extra virgin olive oils [26]. The confusion matrix of the PLS-DA model (Table 3) shows that the sum of the diagonal elements gave a hit rate of 93.8%.

Eight samples from each class were used to construct the model. In the confusion matrix, 3.1% of the samples belonging to groups C and M2 were wrongly classified between the two groups. This may be due to the fact that the amount of ‘Mojo picón’ flavour added to the filling of M2 was too low for all samples to be classified correctly. On the other hand, groups M4 and M8 were successfully predicted with an accuracy of 100%.

### 2.4. Quantification of Sensory Parameters Using E-nose

One way to study the correlation between the data obtained from the E-nose and the tasting panel is Partial Least Squares (PLS) chemometric analysis. This regression model made it possible to quantify and establish prediction models, i.e., from the E-nose and the chemometric model, to obtain sensory analysis values equivalent to those provided by a panel of olive tasters. In this study, the sensory descriptors of the ‘Zapateria’ defect were used.

To obtain the PLS model, 70% of the samples set was split into a calibration set, which was used to calibrate and cross-validate the models. Furthermore, a validation set with the remaining samples (30%) was only used to test the robustness and accuracy of the developed models. Two different PLS models were built, one for each parameter. The $R_{CV}^{2}$ for the models developed for the perceived defect and the overall rating were 0.83 and 0.88, respectively. The RMSECV values (0.78 for the ‘Zapateria’ defect and 1.10 for perceived aroma) were also estimated.

**Figure 2:**
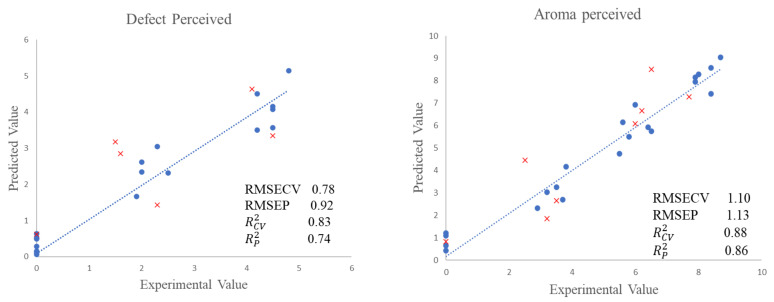
Experimental values against PLS cross-validation predictions (●) and validation set predictions (x) for defect and aroma perceived.

The cross-validation shown in Figure 2 represents the experimental values of the sensory parameters against the predicted values.

In the PLS graph corresponding to the perceived defect, samples with experimental values of zero can be observed. This is due to the fact that at higher concentrations of added ‘Mojo picón’ aroma, the ‘Zapateria’ defect was completely masked. With regard to the graph of perceived ‘Mojo picón’ aroma, the experimental values of zero correspond to the control group samples, to which no aroma was added to the filling.

The predictive ability of a calibration model must be validated with samples not included in the initial calibration. The validation results are also presented in Figure 2 and were very acceptable. The $R_{P}^{2}$ values were 0.74 for perceived defect and 0.86 for perceived aroma, while the RMSEP values were 0.92 and 1.13 for perceived defect and perceived aroma, respectively.

The application of this type of PLS algorithm, in which the E-nose values of perceived defects and aromas in table olives samples are predicted, has been described before: the impact of sterilisation treatments on the effect of cooking in table olives was studied [27].

## 3. Materials and Methods

### 3.1. Experimental Design and Sample Preparation

**Figure 3:**
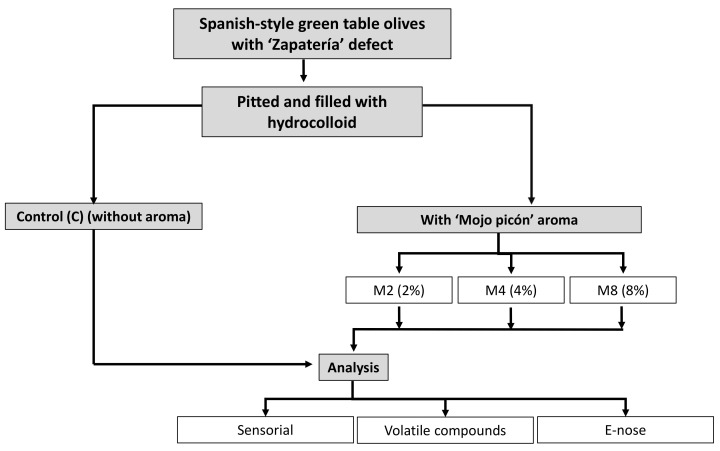
Diagram of the experimental design.

The experimental design is illustrated in Figure 3.

Spoilt Spanish-style green table olives were supplied by a company in southwest Spain. The table olives were first identified and sensory classified by the tasting panel. Then, the olives were pitted for the subsequent stuffing. The different hydrocolloid fillings were prepared with a water base added with 2% sodium alginate, 1% guar gum and 2, 4 and 8% ‘Mojo picón’ commercial flavouring (Neroliane, Grasse, France) according to Sánchez et al. [22]. This flavour is a hot sauce prepared with garlic, cumin, sweet paprika, chilli pepper, sea salt, olive oil and vinegar [28]. The additives were added with 50 mL of water and mixed. All the filling ingredients were food grade. In total, 100 olives were filled manually with a syringe. Four groups of samples were made: (i) table olives stuffed with the hydrocolloid but no ‘Mojo picón’ flavouring has been added. (C); (ii) olives stuffed with 2% of ‘Mojo picón’ flavour (M2); (iii) olives stuffed with 4% of ‘Mojo picón’ flavour (M4); (iv) olives stuffed with 8% of ‘Mojo picón’ flavour (M8). The olives were then soaked for 24 h in a 0.25% CaCl_2_ solution. Subsequently, the olives for each group were placed in jars containing 25 stuffed olives and 100 mL of brine (3% NaCl, *w*/*v*). Finally, the jars were pasteurised at 80 °C for 20 min.

### 3.2. Analyses

#### 3.2.1. Sensory Analysis

A tasting panel formed according to IOC recommendations [1] consisted of eight experts from the research centre CICYTEX (Extremadura, Spain) and the University of Extremadura. For the sensory analysis of the table olives, an evaluation score board was prepared on a structured scale from 1 to 11 points based on perceived positive (‘Mojo picón’) and negative (‘Zapateria’) odors. The used standard of ‘Zapateria’ was cyclohexanecarboxylic acid at different concentrations. The tasting panel was also trained with defected table olives samples given by IOC interlaboratory exchange. The commercial pure aroma of ‘Mojo picón’ was also used as a standard for panel training. The trained panel rated the intensity of the aroma attributes. The results were expressed as average values.

For the sensory analysis, the samples were placed in standard glasses according to IOC recommendations [1]. Each sample consisted of three stuffed olives and 10 mL brine. Eight sample replicates were prepared for each group. In total, 32 samples were analysed.

#### 3.2.2. Analysis of Volatile Compounds

Stuffed green olives were analysed by chromatography for the determination of volatile organic compounds (VOC). The olive samples were crushed and homogenised. A 2.0 g aliquot of each sample was placed in a vial, to which 7 mL of NaCl solution (30% *w*/*v*) was added. A polydimethylsiloxane/divinylbenzene (PDMS/DVB) StableFlex fibre (65 μm, Supelco) was used to sample the volatile compounds. The vials were closed and placed at 40 °C for 30 min according to the methodology described by Sánchez et al. [15] and López-López et al. [29]. Determinations were performed using a gas chromatograph with a triple quadrupole mass spectrometry detector model 456-GC, using a capillary column Agilent DB WAXetr (60 m × 0.25 mm; DI: 0.25 mm). Peaks were identified with the help of the NIST 2.0 MS reference spectral library.

#### 3.2.3. E-nose System

The E-nose used for this work was designed by the research team in Sensory Systems from the University of Extremadura. It consists of a combination of 11 commercially available metal oxide semiconductor (MOX) sensors with global selectivity from the following manufacturers: (i) Bosch BME680: temperature (°C), pressure (hPa), humidity (%RH) and gas measurement (Ω); (ii) Sensirion SGP30: eCO_2_ (ppm), TVOC (ppb), H_2_(2) and ethanol; (iii) ScioSense CCS811: eCO_2_ (ppm), TVOC (ppb) and sensor resistance (Ω); and (iv) ScioSense iAQ-Core: eCO_2_ (ppm), TVOC (ppb) and sensor resistance (Ω). These devices are characterised by the integration of analogue and digital electronics combined with a hot microboard and with the sensing elements on a single chip. The power supply consisted of a +3.7 V lithium battery and communicated via Bluetooth with a mobile phone application. The E-nose measurements took place in two phases: an adsorption phase, in which the sensors are placed in contact with the headspace of the samples for 60 s, and a 30 s desorption phase, in which the sensors are only placed in contact with air, which serves as a reference signal.

### 3.3. Multivariate Data Analysis

The measurements obtained with E-nose had to be processed with chemometric tools for their interpretation. To identify outliers and study the discrimination of potential groups, an exploratory data analysis was first performed using Principal Component Analysis (PCA). This type of analysis reduces the information provided by the E-nose to the minimum number of variables, called Principal Components. Principal Components are linear combinations of the original response vectors. Then, Partial Least Squares Discriminant Analysis (PLS-DA) [30] was performed to identify the components or latent variables (LV) discriminating the most between the different sample groups. A confusion matrix was constructed to deduce cross-validation predictions. From this matrix, the percentage of correct predictions of the sum of the diagonal elements found was calculated.

Another chemometric tool used was Partial Least Squares Regression (PLS), to build quantification models and assess the correlation between E-nose measurements and tasting panel parameters [31]. For this purpose, samples were randomly divided between two sets, one for calibration containing 70% of all samples and a second set for validation containing 30% of the remaining samples. The validation set was used to test the accuracy of the developed models.

The parameters used to assess the accuracy of the models were the root mean square error of calibration (RMSEC), cross-validation (RMSECV) and prediction (RMSEP) and the coefficient of determination for cross-validation ($R_{CV}^{2}$) and prediction ($R_{P}^{2}$). Data analysis was performed using Matlab version R2016b, version 9.1 (The Mathworks Inc., Natick, MA, USA) with PLS_Toolbox 8.2.1 (Eigenvector Research Inc., Wenatchee, WA, USA).

## 4. Conclusions

Spanish-style green table olives stuffed with ‘Mojo picón’ flavoured hydrocolloid improve the commercial category of olives with the ‘Zapateria’ defect. The high concentrations of added flavour meant that the defect was almost imperceptible to the tasters. The main volatile compounds of the ‘Zapateria’ defect, which are cyclohexanecarboxylic acid and pentanoic acid, decreased as the percentage of added flavouring increased. The E-nose discriminates between olives with ‘Zapateria’ defect and stuffed olives with different concentrations of added ‘Mojo picón’ flavouring. The PLS algorithm shows that there is a good prediction between E-nose and sensory analysis. This makes it possible to predict the ‘Zapateria’ defect perceived by the tasting panel.

In the present study, E-nose was successfully used for qualitative purposes and quantitative analysis. Therefore, E-nose can be considered a useful discrimination tool that can be applied to olives stuffed with flavoured hydrocolloids. This device can be used as an aid to the tasting panel and combined with chemometric analysis, can be used to perform rapid, inexpensive, non-destructive and environmentally friendly qualitative analysis.

## Figures and Tables

**Table 1:** Predominantly perceived sensory defects (mean ± standard deviation) of Spanish-style table olives stuffed with flavoured hydrocolloids. Different lowercase letters indicate statistically significant differences according to the intensity of the added flavour between each treatment (Tukey’s Test, *p*-value < 0.05).

	‘Mojo picón’ Aroma	C	M2	M4	M8
**Positive attribute**					
’Mojo picón’	9.7 ± 0.8 a	n.d.	3.1 ± 0.6 d	5.3 ± 0.4 c	7.2 ± 0.6 b
**Negative attribute**					
’Zapatería’	n.d.	5.1 ± 0.5 a	3.5 ± 0.4 b	1.1 ± 0.2 c	n.d.
**Commercial classification**	Extra	2nd. Category	1st. Category	Extra	Extra

C: Spanish-style green table olives without aroma; M2: Spanish-style green table olives with ‘Mojo picón’ aroma at 2%; M4: Spanish-style green table olives with ‘Mojo picón’ aroma at 4%; M8: Spanish-style green table olives with ‘Mojo picón’ aroma at 8%. n.d.: not detected.

**Table 2:** Content of volatile compounds (mean%, *n* = 3) of stuffed olive added with flavoured hydrocolloids. Different lowercase letters indicate statistically significant differences according to experimental treatment for each volatile compound (Tukey’s Test, *p*-value < 0.05).

CAS Number	Volatile Compound	T.R. (min.)	’Mojo picón’ Aroma	C	M2	M4	M8
64-19-7	Acetic acid	2.6	2.6 ± 0.2 a	n.d.	n.d.	n.d.	n.d.
57-55-6	Propylene glycol	5.8	n.d.	19.8 ± 4.5 a	n.d.	n.d.	n.d.
109-52-4	Pentanoic acid	12.1	n.d.	3.1 ± 0.7 a	n.d.	n.d.	n.d.
127-91-3	beta-pinene	15.5	19.7 ± 2.7 b	n.d.	11.8 ± 2.1 a	13.2 ± 2.1 a	11.3 ± 1.3 a
1515-80-6	2,4-Hexadienoic acid, methyl ester	17.8	n.d.	11.2 ± 2.5 a	n.d.	n.d.	n.d.
99-87-6	p-cymene	18.1	18.7 ± 3.4 ns	n.d.	19.2 ± 2.2 ns	21.0 ± 3.2 ns	19.0 ± 2.4 ns
99-85-4	Gamma-terpinene	19.9	24.7 ± 3.5 ns	n.d.	27.4 ± 3.8 ns	27.3 ± 2.7 ns	21.7 ± 3.5 ns
2179-57-9	Diallyl disulphide	21.1	14.6 ± 2.2 b	n.d.	12.1 ± 2.5 a	11.6 ± 1.5 a	14.4 ± 2.4 b
2396-84-1	2,4-Hexadienoic acid, ethyl ester	22.0	n.d.	14.5 ± 3.2 a	n.d.	n.d.	n.d.
98-89-5	Cyclohexanocarboxylic acid	26.5	n.d.	12.7 ± 2.2 c	5.2 ± 1.1 b	2.0 ± 0.5 a	n.d.
93-51-6	Creosol	26.7	n.d.	25.0 ± 3.4 d	5.0 ± 0.8 c	2.4 ± 0.7 a	3.6 ± 0.6 b
88973-62-0	Propyl 2,4-hexadienecarboxylate	26.9	n.d.	9.8 ± 1.5 a	n.d.	n.d.	n.d.
122-03-2	Cuminaldehyde	29.1	6.2 ± 0.8 a	n.d.	9.8 ± 1.1b	9.7 ± 0.9 b	14.3 ± 2.1 c
3913-81-3	2-Decenal, (E)-	29.9	n.d.	3.8 ± 0.5 a	n.d.	n.d.	n.d.
1197-15-5	alpha-terpinen-7-al	31.2	6.8 ± 0.9 b	n.d.	3.5 ± 0.5 a	3.7 ± 0.2 a	6.1 ± 0.4 b
2050-87-5	Allyl trisulfide	31.8	6.8 ± 0.8 a	n.d.	6.1 ± 0.8 a	9.1 ± 0.4 b	9.5 ± 1.3 b

C: Spanish-style green table olives without aroma; M2: Spanish-style green table olives with ‘Mojo picón’ aroma at 2%; M4: Spanish-style green table olives with ‘Mojo picón’ aroma at 4%; M8: Spanish-style green table olives with ‘Mojo picón’ aroma at 8%. RT = retention time. n.d.: not detected.

**Table 3:** Confusion matrix obtained through PLS-DA for discrimination between stuffed olives with flavoured hydrocolloids. Values are expressed in percentage.

Predicted Class				
Real Class	C	M2	M4	M8
C	21.9	3.1	0	0
M2	3.1	21.9	0	0
M4	0	0	25.0	0
M8	0	0	0	25.0

## Data Availability

The authors confirm that the data supporting the findings of this study are available within the article and the raw data that support the findings are available from the corresponding author, upon reasonable request.
